# A protocol for randomized controlled trial on early active flexion versus passive flexion mobilization therapy for Spaghetti wrist injuries

**DOI:** 10.1186/s13018-025-05884-z

**Published:** 2025-05-24

**Authors:** Kishore Vellingiri, Ashwath M. Acharya, Anil K. Bhat

**Affiliations:** Department of Hand Surgery, Kasturba Medical College, Manipal, Manipal Academy of Higher Education, Manipal, 576104 India

**Keywords:** Flexor tendon injury, Zone-5, Spaghetti wrist injury, Flexor tendon rehabilitation

## Abstract

**Background:**

Spaghetti-wrist injuries representing Zone-5 flexor tendon lacerations remain a major challenge for hand surgeons despite the use of careful and meticulous surgical techniques and appropriate rehabilitation programs. Injuries in these regions can lead to adhesion formation, stiffness, and loss of hand function in view of its delicate and complex anatomy. One of the methods to prevent adhesion is the use of active flexion rehabilitation protocols. Its benefits have been shown in numerous reports on zone-2 injuries. However, there is a paucity of reports for this regime in Zone-5 and Spaghetti-wrist injuries.

**Methods:**

This study proposes a double-blind, single-center, randomized controlled trial (RCT) to compare the functional results of two rehabilitation methods following the repair of Spaghetti-wrist injuries. This includes the passive or active flexion therapy regime performed over six weeks. After fulfilling the inclusion criteria, adults aged 18–60 who have presented with spaghetti-wrist injuries will be selected. Patients will receive a comprehensive document outlining the study’s purpose, methodology, and follow-up schedule, which will be a part of the informed consent. 44 patients will be immobilized in a plaster slab and assigned equally to the passive or active flexion group. They will be assessed for primary and secondary outcomes, which include Tang’s criteria, independent digital function, sensory assessment, pinch and grip strength, and Michigan Hand Outcome Questionnaire (MCHQ), each at six and 12 weeks, six and 12 months. The study will follow the SPIRIT guidelines.

**Discussion:**

The proposed RCT compares early active and passive flexion regimes’ functional results in zone-5 flexor tendon injuries. This trial is unique as an active flexion regime was not described earlier in a major comparison study. It will answer the role and possible benefits of a more aggressive early active flexion program. Additionally, the study will give information on patient-reported outcomes and address the incidence of complications in a much longer follow-up of one year.

**Trial registration details:**

The institute ethics committee approval was confirmed for the study (approval No. IEC – 383/2022), and registration for the Clinical Trial Registry of India was completed (CTRI/2023/03/050721).

## Introduction

Zone-5 flexor tendon injuries remain a major challenge for hand surgeons despite using a careful surgical technique and appropriate rehabilitation programs. This is mainly due to adhesions, joint stiffness, persisting nerve injury, and subsequent loss of hand function [1, 2].

There is considerable controversy on the issue of whether to mobilize the tendon early or late after repair. While some advocate for starting physical therapy as soon as possible after tendon repair, others favor longer periods of rest [3, 4]. Zone-5 injuries present challenging problems for surgeons and hand therapists due to their unique and complicated anatomy. Multiple tendons add to the burden of strong adhesions and possible loss of independent motion. Nerve injury repair will be at risk, particularly the median nerve, as it requires fine, meticulous sutures, which need protection and maintenance of low tension with wrist flexion. There is a theoretical risk of disruption of the nerve repair following early mobilization of bunched-up tendon repairs in a tight and edematous compartment. A similar risk exists for arterial repair, particularly the ulnar artery, which is very close to the flexor tendons. However, strict immobilization may also lead to robust adhesions, which may stress the tendon and nerve repair after delayed therapy [4].

Tang and colleagues have argued against starting early mobilization in the first few days, given the increased risk of the inflammatory response, which may exaggerate patients’ pain [5]. According to them and other authors, adhesions usually are not observed before the 10th day, and waiting for at least five days is recommended until the inflammatory phase is over [5–9]. However, the repaired tendons lose tensile strength significantly by two weeks and the excursion [5–7]. Therefore, it is recommended that mobilization be started from the fifth postoperative day onwards [5–9]. Mobilization decreases inflammation and helps reduce the work of flexion and friction [5–7]. As surgical methods and repair strength have increased, place and hold activities, synergistic wrist gliding exercises, and active gliding exercises have all been included in rehabilitation regimens [10]. These include immobilization regimens, place-and-hold exercises, and passive and active motion regimens [2, 3, 10]. Subsequently, many authors started studies on early active-motion protocols. As the evidence became clearer with improved functional results following early mobilization protocols, perhaps due to more efficient tendon gliding with minimized adhesions, the role of prolonged immobilization decreased. Later studies showed that tendons can heal without adhesion. In the only randomized study described for zone-5 injuries, Udayaraj et al. compared the Kleinert’s with a passive motion regime and observed the former to show better clinical improvement [11]. However, active flexion was not tested in these regimes, and the assessment duration was three months; hence, nerve injury and long-term assessment information were unavailable.

The main concern with active-motion programs is the risk of tendon rupture. However, the clarity on outcomes in the literature is lacking in view of variables like type and level of injury, technique of repair, and rehabilitation regimes [2, 8, 9, 12, 13]. Studies have also reported problems with passive mobilization protocols such as adhesions, joint contractures, and extensor lag [14]. For zone-5 flexor tendon injuries, no studies have compared the benefits of passive mobilization with early active motion therapy. In this regard, we propose conducting a randomized control trial (RCT) with the aim of comparing the functional results and complications of early active motion therapy versus passive mobilization for zone-5 flexor tendon injuries.

## Methods and analysis

### Study design

**Fig. 1:**
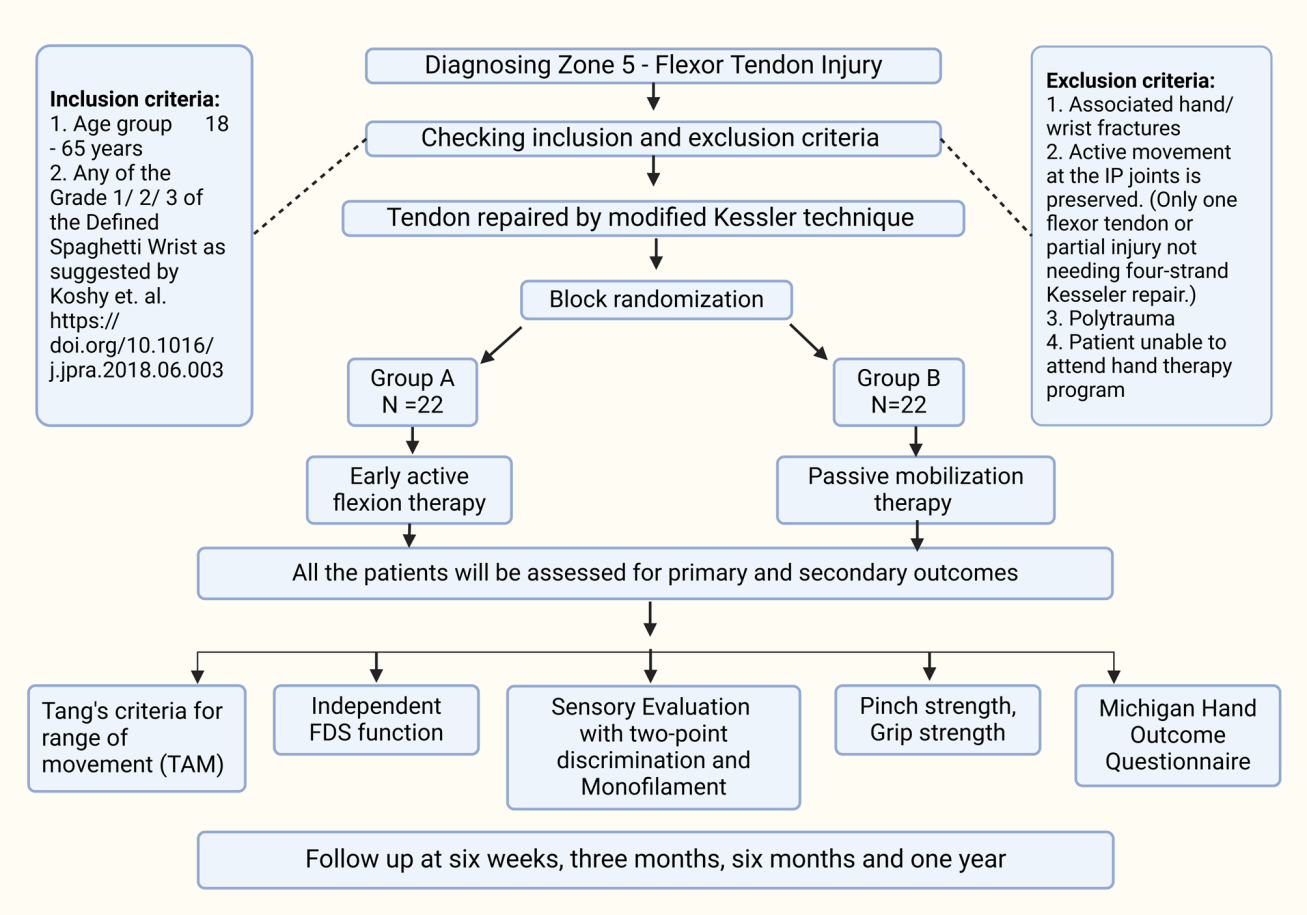
Shows the flow chart for the randomized control trial program. TAM– Total Active movement, FDS– flexor digitorum superficialis

The double-blind RCT will be conducted at a single tertiary referral center, comparing the functional results and complications after using two different rehabilitation programs in patients presenting to the Trauma Centre and Outpatient Department of Hand Surgery with zone-5 flexor tendon injuries. The timeline for the trial procedure is demonstrated in Fig. 1. Patients presenting from March 2023 to February 2026 fulfilling the inclusion criteria will be explained about the procedure, the risks and advantages involved, the type of subsequent rehabilitation, and evaluation by the trained instructors. All the patients will be required to provide written informed consent, and the data collected will be documented and stored for the study. The enrollment evaluation checklist and the informed consent obtained are provided in supplementary files 1 and 2 each. The protocol is prepared as recommended in the guidelines laid out in the Standard Protocol Items: Recommendations for the Intervention Trials (SPIRIT) [15].

### Study population

All patients aged 18–65 years qualifying for the inclusion criteria (vide infra) are considered for the trial. Comprehensive information encompassing enrollment, details of the surgery, plan of immobilization, associated benefits, risks, and complications, type of rehabilitation protocol, methods of evaluation during the follow-up, and assigned person of contact for queries and emergency events will be provided. The patient’s privacy will be protected, and those unwilling to continue the trial program will be allowed to exit the study unconditionally. Patients will be informed that they will be included in the active or passive flexion mobilization regime. Patients will be counseled to provide written informed consent.

**Inclusion criteria**:

1. Age group: 18–65 years.
2. Any of Grade 1/ 2/ 3 of the Defined Spaghetti Wrist as suggested by Koshy et al. [3]

**Exclusion criteria**:

1. Associated hand/ wrist fractures.
2. The active movement of the digit is preserved at IP joints (Only one flexor tendon for finger or partial injury not requiring four-strand Kessler repair).
3. Polytrauma.
4. Patient unable to attend hand therapy program.

### Randomization and blinding

Forty-four patients who fulfill the study’s inclusion criteria and have signed the informed consent form will be randomly assigned in a 1:1 ratio of 22 each, using block randomization for either the early active motion group or the passive motion group. This block randomization will be created using a computer-generated randomized controlled table. Randomization will be done 24 h before the surgery. An opaque sealed envelope with sequential numbers will hold the allocation sequence. The sole information on this envelope’s label will be its unique ID number, which is numbered progressively and includes study details. To ensure a blind and identical appearance at the time before surgery, an independent resident not involved in the treatment will receive the envelope. They will then prepare the patient for an active or passive flexion mobilization program (Table 1). The active versus passive mobilization protocol is tabulated in Table 1. The surgeons, hand therapists, and data assessors tracking the patients are thus blinded by this approach, which will be maintained until the end of the trial period.

**Table 1:** Table showing the regime followed for the early active and the passive mobilization groups

Weeks	Early protected active	Passive mobilization
5th day	Dorsal thermoplastic splint with wrist 10^0^ flexion, MCP 40^0^ flexion, and IP full extension (Wrist immobilized till 6 weeks)	Dorsal thermoplastic splint with wrist 10^0^ flexion, MCP 40^0^ flexion, and IP full extension. (Wrist immobilized till 6 weeks)
Day 5–12	25% excursion (active flexion) of the affected digits at IP and MP joints (Remove the Velcro strap at the digital and palmar level) The patient is instructed to flex only 25% of the range possible and extend the fingers until full extension at IP joints within the splint. (20 repetitions/10 times a day of active flexion and extension exercise per hour)	Full passive flexion of the affected digits at IP and MP joints (Remove Velcro strap at digital and palmar level) The patient is instructed to flex the finger passively with the other hand. Fingers actively extended within the splint as far as possible. (20 repetitions/10 times a day of passive flexion and active extension exercise per hour)
Day 12–21	50% excursion (active flexion) of the affected digits at IP and MP joints. (Remove the Velcro strap at the digital and palmar levels) The patient is instructed to flex only 50% of the range possible and extend the fingers until full extension at IP joints within the splint. (40 repetitions/ a day of active flexion and extension exercise) Ultrasound scar massage Tendon mobilization/gliding exercise and full ROM exercise started at 3 weeks.	Full passive flexion of the affected digits at IP and MP joints (Remove the Velcro strap at the digital and palmar levels) Flex the finger passively with the other hand. Fingers actively extended within the splint as far as possible. (40 repetitions/ a day of passive flexion and active extension exercise) Ultrasound scar massage Tendon mobilization/gliding exercise and whole ROM exercise started at 3 weeks.
Week 3–5	Splint modification - wrist brought to the neutral position. Remove the splint hol, d the wrist in neutral with the other hand, and encourage full active motion flexion and extension of IP and MP joints. Reapply the splint after exercise therapy. For the passive mobilization group, passive full flexion and active hold are started, followed by full active movement of flexion and extension as the weeks progress. (20 repetitions/ 10 times a day of active flexion and extension exercise per hour) At the end of the fourth week, full active motion range should be the goal (full extension at IP and MP joints) Low-intensity ultrasound scar massage (with intensities of 1 MHz, pulsed, 0.5 W cm-2) will be applied. Electrical stimulation of muscles in the hand for neurotmesis of the ulnar or median nerve (hypothenar/ thenar/ dorsal side for interossei) will be performed. Current type - Galvanic current () / Pulsed alternating current (AC) after re-innervation Pulse/cycle duration - >100 ms (GC) or 0.1-1 ms (Faradic current) Amplitude– as tolerated by the patient or till visible contraction observed Duration − 3 cycles of 30-30-30 contractions (Galvanic current), < 30 s (AC) Session frequency − 3–5 times/week, for 8–12 weeks approximately [16]	
Week 6	Wrist mobilization and electrical stimulation of long flexors, thenar, and hypothenar are started. Progression to full range of movements of wrist and fingers	
Week 8	Blocking and light resistance exercises, full resistance is prohibited, splint is removed and worn only at night. Activities of daily living (ADL) are not yet advised. Introduction of the static splinting program to get at the interphalangeal joint (IP) extension in cases of flexor stiffness and flexion strapping program for extensor stiffness at IP joints Splinting for nerve injury will be added, which includes a knuckle bender splint if ulnar nerve injury is present and a first web spacer splint to keep the thumb in palmar abduction and extension at the CMC joint in median nerve injury	
Week 10	Stretch board exercises for IP and metacarpophalangeal (MP) joint stiffness and aggressive resistive mobilization started.	
Week 12	Desensitization exercises and activities of daily living started	

### Interventions

All patients in both groups will undergo zone-5 flexor tendon repair using the four-stranded modified Kessler technique, interrupted epineural nerve repair, and anastomosis of the principal artery.

## Procedure

### Preoperative management

The Department of Hand Surgery proforma will be used to gather baseline demographic data, which will be used to screen patients for inclusion and exclusion criteria. A preoperative clinical assessment will assess several factors that may impact the patient’s outcome following surgery. A radiograph of the affected extremity will be taken to rule out fractures. All patients will get preoperative antibiotics with tetanus prophylaxis to prevent infections. Every patient will have routine operational investigations, such as complete blood counts and serology testing, in accordance with hospital and departmental policy. Following a clear and concise explanation of the trial, written informed permission will be obtained from the participants.

### Surgical procedures

All patients will undergo surgery under regional or general anesthesia and a tourniquet. All the repair procedures will be done under loupe or microscope magnification. Structures will be repaired in the serial order of deep to superficial and far corner to near. Flexor tendons will be repaired using a modified Kessler repair involving four-strand core sutures using 3 − 0 polypropylene with the knot at the cut end. 8 − 0 or 9 − 0 nylon is used to repair the median/ ulnar nerves and radial/ulnar arteries.

### Follow-up

Patients in both groups will be followed up each at six weeks, three, six, and 12 months.

### Outcomes

Primary outcome and Secondary outcomes.

Patient-related variables:

1. Age.
2. Gender.
3. Co-morbidities.
4. Personal habits.
5. Preoperative mobility.
6. Patient injury data will be sub-grouped based on the classification of Koshy et al. They will be divided into three grades based on the lacerated structures, laceration type, and repair type [3].

Primary outcome: Tang’s criteria (Total active movement) [17].

Secondary outcome:

1. Independent FDS function.
2. Sensory assessment.
3. Pinch strength.
4. Grip strength.
5. MCHQ (Michigan Hand Outcome Questionnaire) [18]– Functional outcome.
6. To look for causes of motor passivity that could influence the outcome, we propose using the Kinesiophobia Causes Scale (KCS). It is observed that fear of movement, considered to be a part of an individual’s personality, could be a major limitation to motor activity. This type of behavior is described as kinesiophobia and can be documented using this scale [19].

### Postoperative **care**

Patients in both groups will receive a dorsal thermoplastic splint on day five and start on either the active or passive mobilization program. Table 1 displays the protocol for mobilization.

### Safety **monitoring and adverse events reporting**

Adverse events will be documented and supervised by the principal investigator and promptly reported to the IEC within 24 h. In the event of a complication, treatment expenses will be covered by the investigating department and institution.

Benefits: After a hand therapy program is completed, the study will assist in determining the correlation between variables and their impact on the outcome of zone-5 flexor tendon restoration. Once established, corrective actions and rehabilitation can be used to further mitigate the impacts of variables and achieve a beneficial outcome in these injuries.

### Sample **size calculations**

At a 5% level of significance with 80% power and an effect size of 0.3, the minimum sample size (per group) required for a two-group repeated measures study design involving one primary quantitative outcome measured across four time points [six weeks, three months, six months, one year] (correlation among repeated measures is clinically presumed to be 0.3), was 44, i.e., 22 per group.

The sample size is computed using G*Power 3.1.9 and based on the formula mentioned below.

Formula.

$$n=\frac{2}{{{\delta ^2}}}\left[ {\mathop \sigma \nolimits_{b}^{2} +\frac{{{\sigma ^2}}}{k}\left[ {1+\left({k - 1} \right)\rho } \right]} \right]{\left({{z_{{\raise0.7ex\hbox{$\alpha $} \!\mathord{\left/ {\vphantom {\alpha 2}}\right.\kern-0pt}\!\lower0.7ex\hbox{$2$}}}}+{z_\beta }} \right)^2}$$

Where,

$\mathop \sigma \nolimits_{b}^{2}$: Between group variance

${\sigma ^2}$: Within group variance

$\rho$: Intra class correlation coefficient

$$\delta $$: Effect size

*K*: Number of repeated measures

$$\alpha $$: Significance level

$$1 - \beta $$: Power

### Statistical **analysis**

An MS Excel spreadsheet application will code and record the data. Programming or SPSS v23 will be utilized to analyze the data. For continuous variables, descriptive statistics will be explained as means/standard deviations, IQR/ medians, and percentages/ frequencies; categorical variables will take the form of a chi-square test.

Where applicable for data visualization, data will be displayed graphically using pie or bar charts for categorical data and box-and-whisker plots, histograms, and column charts for continuous data. The independent sample “t” test will be utilized for group comparisons for continuously distributed data, and repeated measures ANOVA (Analysis of Variance) will be employed to evaluate the results across time and among the groups.

### Data management

A distinct number will be given to each patient, and this number will be used to record all clinical information. Perioperative assessment and examination will be the responsibility of the consultant hand surgeon and the clinical resident. The research associate will oversee the postoperative evaluation, and the associate hand surgeon will be blinded. The research associate and clinical resident will conduct routine follow-ups for a year after surgery. Using the designated computer, the Principal Investigator will oversee data entry and storage. After data gathering, an electronic database will be created and backed up. After this study, all raw data will be kept in a medical recording room for at least three years.

### **Data** monitoring **and auditing**

The principal investigator in charge of the research study will ensure and confirm that each participant has signed an informed consent form before beginning the study. He/she will also ensure that the research adheres to the study protocol, upholds all pertinent laws, and collects accurate and comprehensive data and observations. The data monitoring committee, composed of three experts in hand therapy, occupational therapy, and statistics, will perform audits via phone calls or routine interviews. They also maintain the power to examine patients at random on any occasion. The audit procedure will be completed without the involvement of the investigators’.

### Strategies **to improve adherence to protocol**

To conduct the recruitment process, we will obtain informed consent and follow-up and standardize the evaluation scales to assess outcomes; all study members will undergo an informative, professional training program.

### **Ethics** and **dissemination**

The Institute Ethics Committee endorsed the trial (approval No. IEC – 383/2022). The study design and informed consent documents addressed the minimal potential risks of this RCT. Each candidate fulfilling the inclusion criteria will provide written informed consent before the enrolment. The trial is registered at the Clinical Trial Registry of India (CTRI)(registration number: CTRI/2023/03/050721). The data from this trial and the CTRI registration and IEC approval form will be submitted together. The principal investigator will pay for any secondary revision procedure. The results of this trial will be shared and published in academic forums and peer-reviewed research journals. Relevant authorities, including trial registries and the institute ethics committee, will be informed of protocol modifications (e.g., interventions, sample size changes, outcomes, and analyses).

### Patient **and public involvement**

The planning, execution rep, sorting, or distribution of the study will not involve patients or members of the public.

## Discussion

The proposed novel study of a double-blind, randomized trial conducted at a single center, comparing the functional results of early active motion therapy with passive mobilization in zone-5 flexor tendon injuries, is unique and has not been described earlier. It will answer the role and possible benefits of a more aggressive early active mobilization program. Additionally, the study will give information on patient-reported outcomes and address the incidence of complications.

How successfully a flexor tendon heals depends on several factors, including the type of injury, any concurrent nerve damage, the surgeon’s experience, treatment methods, and postoperative care [2, 8, 9, 20]. Several different postoperative mobilization techniques exist, from rigid immobilization to early, protected finger movement. However, the description of the rehabilitation program after flexor tendon surgery in zone-5 is not well documented in the literature [21]. The only regime followed for injury in these regimes was a form of early protected passive range movements. The majority of the studies reported acceptable function with early mobilization, with a higher complication rate being observed in more severe tendon and nerve injuries [2, 10, 22–24].

In one of the earliest reports on a comparison study between active and passive mobilization regimes for zone-5 flexor tendon injuries, Panchal et al. reported results observed in two each cadaver and patients [4]. The mean excursion of the FPL, FDP, and FDS will be calculated at three time periods of 10 days, three, and six weeks. The authors found an ‘increased’ excursion in patients who followed the active flexion mobilization program [4]. Although early active mobilization has been advocated since then, true active flexion regimes have been described in very few studies, and no comparisons have been made between different methods [3]. Yii et al. did a prospective study in zone-5 flexor tendon injuries and introduced an active extension and active flexion regime [25]. A series of 52 patients with an average of 10 months follow-up showed independent FDS activity in 66%, with 90% achieving good to excellent results concerning range of motion [25]. However, this series also included non-Spaghetti injuries, and ‘Spaghetti injury’ was attributed as a risk factor for adverse results. Udayraj et al. have compared Kleinert’s protocol (which involves the active extension of the digit program) with a passive range of movement regime in a randomized study involving 30 patients [11]. They demonstrated a better clinical improvement in Kleinert’s protocol when compared to the passive mobilization group [11]. However, only the total active movement and grip strength were measured, and the study period was a limited duration of 12 weeks. The current proposed study addresses all these issues as we plan to evaluate multiple parameters over a one-year period.

In the last two decades, significant advances have helped in the understanding of flexor tendon healing, resulting in better techniques in exposure, repair, and rehabilitation [2, 26, 27]. Much emphasis has been put on early active flexion of the injured digit as the new repair techniques have permitted a greater load on the repair site [5]. However, most of this research work has been done for the region of zone 2. The current authors believe this can also be applied to Zone 5. However, it must be compared with the existing passive mobilization protocol to confirm its benefits.

In a systematic review of outcome measures used for zone-5 injuries, Cardozo et al. observed a lack of consistency in the outcome measures used for assessment [10]. They observed eight measures used in different studies, with the TAM being the most common [10]. We have used the TAM with Tang’s criteria to document quality improvement. The sensory-motor recovery has also been added, as suggested by Cardozo et al. We have also introduced the patient-reported outcome measure of MCHQ to make it a more comprehensive evaluation.

The limitations of this research are primarily associated with it being conducted within a singular institutional framework. Nevertheless, it will ensure uniformity in the management of the patients. To summarize, the purpose of this research is to ascertain the minimal duration of immobilization required to ensure sufficient healing of a flexor tendon repair in zone 5 under the condition that a standardized rehabilitation protocol is implemented.

## Data Availability

No datasets were generated or analysed during the current study.
